# Relationships among Actual Motor Competence, Perceived Motor Competence, and Health-Related Fitness in College-Aged Males

**DOI:** 10.3390/sports8120158

**Published:** 2020-12-02

**Authors:** Samantha Moss, Erik Lind, Rick Ferkel, Peter McGinnis, Larissa True

**Affiliations:** 1 Department of Kinesiology, University of Texas at Arlington, Arlington, TX 76019, USA; Samantha.moss2@mavs.uta.edu; 2 Kinesiology Department, State University of New York at Cortland, Cortland, NY 13045, USA; erik.lind@cortland.edu (E.L.); peter.mcginnis@cortland.edu (P.M.); 3 College of Health Professions, Central Michigan University, Mt Pleasant, MI 48858, USA; ferke1r@cmich.edu; 4 Department of Kinesiology and Dance, New Mexico State University, Las Cruces, NM 88011, USA

**Keywords:** skill proficiency, fitness, college students, motor skills, physical activity, perceived competence

## Abstract

Actual motor competence (MC), perceived motor competence (PMC), and health-related fitness (HRF) exhibit a dynamic and reciprocal relationship in child populations, but little is known about the nature of these relationships in young adulthood. The purpose of the study was to assess these relationships in a sample of college-aged males. A total of 55 participants enrolled in an undergraduate Kinesiology course completed the study. Perceived motor competence (PMC) was assessed with the Physical Self-Perception Profile questionnaire; MC was assessed using maximum throw and kick speed and maximum jump distance; HRF was assessed with a two-minute push-up test, two-minute sit-up test, and the Multistage 20-m Shuttle Run Test. Pearson’s bivariate correlations were calculated to assess relationships among PMC total score, MC scores, and HRF scores. Two separate indices were calculated to create composite total MC and total HRF scores used for subsequent analyses. Significant correlations were found between PMC total score, MC index, and HRF index. Multiple linear regressions were used for analyzing predictive measures for HRF and PMC scores. From the two regression models, significance varied among total MC scores, PMC scores, and HRF individual measures. These findings may suggest that relationships among MC, HRF, and PMC strengthen over developmental time in young adult males.

## 1. Introduction

Many factors contribute to the capability of an individual to perform skilled movement. Motor competence (MC) is a global term that describes an individual’s level of ability to perform gross fundamental motor skills such as throwing, kicking, jumping, and running [1]. MC has been studied fairly extensively in child populations in relation to perceived motor competence (PMC) and health-related fitness (HRF) [2,3,4,5,6]. PMC is defined as an individual’s subjective perception of their propensity for performing motor skills and physically-demanding activities [7]. HRF is the capacity of an individual to perform physical work, and is commonly classified in the domains of cardiovascular endurance, muscular endurance, muscular strength, body composition, and flexibility [8].

In 2008, Stodden and colleagues [9] proposed a theoretical model that hypothesized dynamic relationships among MC, PMC, HRF, and physical activity at different points throughout childhood. The crux of this model is that relationships among MC, PMC, HRF, and physical activity strengthen over developmental time, and children who are motorically competent have high perceptions of their motor ability, are sufficiently fit and physically active, and are more likely to continue such behaviors into adolescence, and eventually, adulthood. A considerable amount of research has confirmed the various hypotheses generated in Stodden’s model [9] at early [3,10,11,12,13], middle [3,5,13,14], and late childhood [3,10,13,15,16,17], and to a limited extent, into adolescence [18,19,20]. In general, research in adolescent populations suggests that their physical activity levels are significantly influenced by HRF and PMC; specifically, higher physical activity levels are achieved in adolescence when individuals are highly fit and have high perception of motor competence in childhood [18,20]. MC and HRF are also significantly associated in adolescence, further creating a need to explore these interrelationships in older populations [19]. A recent meta-analysis indicated a moderate to strong relationship between HRF and MC from early childhood to young adulthood, while acknowledging that other factors not assessed (possibly PMC) could have contributed to the strength of this relationship, warranting future research [21]. On the other hand, a recent study has challenged these theories by finding no significant relationship between PMC and MC in their sample of children [22].

Although Stodden and colleagues [9] contend that such relationships should theoretically exist in adulthood, there is little empirical evidence to support such claims. Silva et al. [23] found significant associations within their MC constructs only in their young adult male population. Similarly, Sackett and Edwards [24] found measures of MC and PMC to be influential factors of cardiovascular fitness in both male and female college-aged participants. Given that the models that were explored demonstrated a better fit for females in the sample, the authors encouraged future research on male college-age participants [24]. Previously, two studies examined the hypothesized relationships presented in Stodden’s model in a young adult population, but these were limited to only the MC/HRF relationship. Results indicated that MC predicted 79% of the variance in HRF in young adults [25,26], which did lend credence to the notion that the relationship between MC and HRF will strengthen over developmental time; however, neither PMC nor physical activity were examined in these studies. While longitudinal research is necessary to confirm the existence and directionality of these developmental trajectories in adulthood, cross-sectional, descriptive research can serve as an initial line of inquiry in the realm of correlates of skilled movement in young adult populations. The purpose of this study was to assess the relationships among MC, PMC, and HRF in a college-aged male population. Our hypotheses were twofold: (1) We hypothesized that significant, positive relationships would exist among individual MC and HRF scores and PMC total scores; and (2) We hypothesized that calculated HRF and MC indices would predict PMC scores.

## 2. Materials and Methods

### 2.1. Participants

A total of 57 male participants from a university in the northeast region of the United States were recruited to participate via an undergraduate Kinesiology course with an associated laboratory session. Participants were recruited from their scheduled lab session, each of which held approximately 20 students. Students were given the option to participate in the study or complete the regularly scheduled lab assignment for the week in which testing was scheduled. Students that chose to participate in the study earned the same number of points that could have been earned by completing the lab assignment. Exclusion criteria included having sustained an orthopedic injury in the last six months or the presence of any known cardiorespiratory conditions. A total of 55 men completed all measures for the study (demographics, anthropometrics, PMC, MC, and HRF). Two students who were absent the day of testing opted to complete the lab assignment rather than participate in the study. Prior to the onset of the study, participants completed an informed consent and approval was obtained by the university’s Institutional Review Board (document protocol approval #171837).

### 2.2. Procedures

Participants completed the informed consent, the Physical Self-Perception Profile, and had their height and weight taken by a member of the research team during a previously scheduled class time one week prior to the testing day. The MC tests and the HRF tests, respectively, were completed in a large indoor recreation area that was easily accessible for all participants. Four separate data collection sessions were conducted over the course of a single day, with 12–15 participants in each collection session. Upon arrival to the recreation area, all participants performed a 10-min, unstructured (but required) warm-up that consisted of jogging around the area, stretching, and/or throwing/kicking with the available equipment. MC testing took place immediately following the warm-up period. Throwing speed was measured first, followed by kicking speed. Participants completed the jumping task with a member of the research team while waiting to complete the kicking and throwing measures. After all participants completed the three MC tests, the HRF portion took place. The push-up test was conducted first, followed by the sit-up test. Both tests had alternating turns for their partners, which allowed for adequate rest time between the MC assessments and push-up assessments. If participants requested a longer rest time, it was granted. Last, the Multistage 20-m Shuttle Run Test was conducted.

### 2.3. Measures

Age and sex were self-reported on a brief survey attached to the informed consent. Participants’ standing height and weight were directly measured using a portable stadiometer (Chadar HM 200P, Portstad, Taiwan) and a portable scale (Omron Hn-286, Singapore), respectively. Height was measured to the nearest 0.1 cm and weight was measured to the nearest 0.1 kg.

The revised version of the Physical Self-Perception Profile [27] was used to assess PMC and was distributed to participants one week prior to MC and HRF testing. This Likert-type questionnaire is designed to assess participants’ perceptions of their performance on certain motor and fitness tasks. There are five subscale items including sports competence, physical condition, body attractiveness, physical strength, and physical self-worth. Total subscale scores ranged from 6 to 24 (maximum total score = 120) with a higher score indicating a higher (i.e., more positive) self-perception. For this study, the total overall score was retained for analysis (minimum score = 30, maximum score = 120). Sample statements included: “I feel extremely satisfied with the kind of person I am physically”, “I am sometimes slower than most when I learn a new sports-related skill”, and “I make certain I take part in some form of regular, vigorous physical exercise”. For each statement, participants were given the option to choose “very untrue” (1 point), “somewhat untrue” (2 points), “somewhat true” (3 points), or “very true” (4 points).

Gross MC was assessed in a single testing session using product (outcome) scores of three tasks: throwing, kicking, and jumping; this approach has been used in other studies to examine MC in a young adult population [25,26]. Participants were instructed to throw a tennis ball with their dominant hand “as hard as possible” to a wall with no target from a distance of approximately 9 m. A member of the research team used a radar gun (Bushnell Velocity Speed 101911, Bushnell, Overland Park, KS, USA) to measure speed in kilometers per hour by standing 1 m behind the participant at a 45-degree angle. Participants completed five trials of the throw and the maximum speed among the five trials was retained for analysis. Maximum speed was used to reflect the participants’ best performance rather than an average of all five trials. A similar approach was taken to assess kicking speed. Participants were instructed to kick a soccer ball (regulation size 5 ball, 420 gr, 68 cm, inflated to approximately 11.0 psi) with their dominant foot “as hard as possible” using a self-directed approach (e.g., either a running approach or no approach could be used based on the discretion of the participant). Maximum kicking speed among 5 trials was retained for analysis. To assess jumping, participants stood behind a line next to a tape measure and were instructed to “jump as far as possible” while landing on two feet with no approach. A member of the research team marked where the participant’s heel that was closest to the starting line landed and recorded the distance jumped. The maximum jump distance across 5 trials was retained for analysis. The final “jump” variable was divided by the height of the participant to create a standardized variable (e.g., jump distance/height).

Participants completed three tasks to assess HRF: push-ups, sit-ups, and the Multistage 20-m Shuttle Run Test (also called the Beep Test or PACER test). The push-up test and sit-up test followed the same protocol used in the U.S. Army Physical Fitness Test [28]. A timed two-minute push-up test was used to assess upper-body strength and endurance. A member of the research team who was a certified personal trainer instructed and demonstrated to all participants how to properly execute a non-modified push-up; the personal trainer also gave specific instructions and a demonstration on the difference between a properly and improperly performed push-up so that the partner counting the push-ups would know when to not give credit for a push-up. Following the demonstration, participants formed into groups of two; one partner counted the number of correctly performed push-ups while the active partner completed as many push-ups as possible. The test was self-paced, meaning that participants could stop and rest and then, resume again at any point during the testing period; however, if a push-up was executed with improper form, it was not counted. Additionally, four members of the research team trained in proper push-up protocol observed the testing and interjected when necessary. Verbal encouragement was given by the research team and the non-active partner throughout the tests. After the first partner completed their push-ups, the partners switched roles and the test was repeated.

Next, abdominal strength and endurance were assessed using a two-minute sit-up test [28]. A member of the research team who was a certified personal trainer instructed and demonstrated to all participants how to properly execute a sit-up; the personal trainer also gave specific instructions and demonstration on the difference between a properly and improperly performed sit-up so that the non-active partner would know when to not give credit. Participants were instructed to perform as many sit-ups as possible. Still in pairs, one participant performed the test while the other participant was actively assessing the sit-up form of their partner and counted the amount completed correctly. A visual check of sit-up form validated that participants’ hands were placed across their chest and the shoulders were lifted off the ground while the non-active partner secured both feet to the ground holding the ankles. The test was self-paced, meaning that participants could stop, rest, and resume at any point during the testing period; however, if a sit-up was executed improperly, it was not counted. Additionally, four members of the research team trained in proper sit-up protocol observed the testing and interjected when necessary. Verbal encouragement was given by the research team and the non-active partner throughout the tests. After the active participant completed the test, the partners switched roles and repeated the test.

The final measure of HRF was the Multistage 20-m Shuttle Run Test (i.e., “laps ran”) [29], which was used to measure cardiorespiratory endurance. Still in pairs, participants lined up on one end of a large court with one partner actively participating and the other partner recording. The indoor court was a poured, rubberized floor that is appropriate for a variety of activities (e.g., running, floor sports, ball sports). A line 20 m from the starting line was indicated to participants, who were instructed to run to the opposing line after hearing a “beep”. Once participants crossed the opposing line, they were instructed to wait for the next “beep” before running back to the original starting point. This process continued with increasing frequency of beeps. The initial minimum running velocity was paced at 8.5 km/hr^−1^ and increased by 0.5 km/hr^−1^ each minute. When participants were further than 2 m from the line when the beep sounded (or when participants self-selected to stop running), their test was completed and the number of total laps ran was recorded. Verbal encouragement was given by the research team and the non-active partner throughout the test. After the active participant completed the test, the partners switched roles and the test was repeated.

### 2.4. Data Analysis

All data were analyzed using IBM SPSS version 25.0 (Armonk, NY, USA: IBM Corp). Prior to data analysis, all variables were assessed for normality of distribution using a Shapiro–Wilk test. Means and standard deviations were calculated for age, height, weight, and BMI. Likewise, means and standard deviations were also calculated for MC, HRF, and PMC measures. An MC index was created as an “overall” MC score for each participant by averaging maximum scores on throwing, kicking, and jumping. Similarly, an HRF index (overall HRF score) was calculated for each participant by averaging the number of push-ups, sit-ups, and laps completed. A series of Pearson’s bivariate correlations were calculated on individual variable scores (e.g., throwing, kicking, jumping, sit-ups, push-ups, laps, PMC score). Pearson’s bivariate correlations were also calculated for the MC and HRF index scores and PMC scores. Lastly, two multivariate regressions were calculated based off significant findings from the correlation analyses. The first regression analysis used PMC scores and the MC index to predict HRF index scores; the second analysis used individual HRF measures to predict PMC scores.

## 3. Results

Means and standard deviations of physical characteristics and outcome measures are presented in Table 1. On average, participants were 20.2 years old, weighed 80.1 kg, and were 1.78 m tall with a BMI of 25.3 kg/m^2^. Most were right-hand (89.1%) and right-foot (85.5%) dominant. Results from the Shapiro–Wilk test indicated that all outcome variables were normally distributed (*p* > 0.05). Table 1 also shows the means and standard deviations of the MC variables used for analyses (throw max, kick max, jump max/height, MC index), HRF variables used for analyses (laps completed in Multistage 20-m Shuttle Run Test, push-ups, sit-ups, HRF index), and PMC total score.

Pearson’s bivariate correlations among individual MC and HRF variables and the PMC total score are presented in Table 2. For MC variables, results indicated that significant, moderately strong correlations existed between kicking and throwing (*r* = 0.352, *p* < 0.01); jumping and throwing (*r* = 0.467, *p* < 0.01); and jumping and kicking (*r* = 0.433, *p* < 0.05). Throwing was not significantly correlated with any other variable outside of MC measures. Within HRF variables, sit-ups and laps ran (*r* = 0.509, *p* < 0.01) were significantly, moderately correlated, whereas sit-ups and push-ups were not (*p >* 0.01). Jumping and push-ups were both significantly correlated to PMC score (*r* = 0.350, *p* < 0.05, and *r* = 0.410, *p* < 0.01, respectively).

A second series of Pearson’s bivariate correlations was conducted for the three main constructs of the study: MC index, HRF index, and PMC total score (Table 3). The only significant relationship among the constructs was for PMC score and HRF index (*r* = 0.355, *p* < 0.05).

A multiple linear regression was conducted (Table 4) and revealed that MC index scores and PMC total scores accounted for significant amounts of variance in HRF index scores (R^2^ = 0.167, *F* = 4.56, *p* = 0.015).

A second multiple linear regression was conducted (Table 5) with individual constructs of HRF (push-ups, sit-ups, and laps completed) labeled as predictors to PMC total score. The analysis revealed 18.3% of variance was shared among HRF measures and PMC total score (R^2^ = 0.183, *F* = 3.37, *p* = 0.027).

## 4. Discussion

The purpose of this study was to investigate relationships among MC, HRF, and PMC in male Kinesiology students (*n* = 55). Compared to a similar cohort of college-aged males, our sample demonstrated poorer throwing, kicking, and jumping [26]. Lower scores could indicate less time spent practicing these skills in young adulthood. Additionally, our sample demonstrated poorer cardiovascular endurance, but greater upper body strength/endurance, and similar abdominal strength/endurance in comparison to a recent sample of college-aged males who completed the same battery of HRF tests [24].

Significant correlations among the individual MC scores, individual HRF scores, and PMC scores existed (Table 2). We contend that the individual relationships between the two individual MC measures to two of the three HRF measures is an important one. It is possible that individuals who are highly skilled in various motor tasks such as kicking and jumping were exposed to these activities as children and this exposure promotes their subsequent (e.g., young adulthood) levels of HRF [9]. Past history of sport participation (for example, soccer and basketball) could be significant contributors to these associations. One longitudinal study examined children’s (*n* = 1045) motor skill proficiency in comparison with their adolescent (grades 10 and 11; *n* = 244) object control skills, locomotor skills, and cardiorespiratory fitness [30]. Their findings suggested that childhood object control was the strongest predictor of cardiorespiratory fitness in adolescence. Although our examination was not longitudinal, we found that cardiorespiratory fitness (e.g., laps ran) was significantly related to only jumping and sit-ups, of which laps ran and sit-ups exhibited the strongest relationship. Though our findings in this lens differ from Barnett et al. [30], it is reasonable that jumping and our measure of cardiorespiratory fitness (e.g., running) would be related because they are both categorized as locomotor skills.

The sit-up variable was only significantly related to cardiorespiratory endurance; there were no other significant relationships within HRF measures. This contradicts Stodden et al. [25], who found weak but significant correlations between curl-up and throw, kick, and jump (*r* = 0.48, *r* = 0.49, *r* = 0.59, respectively). Our lack of findings here could be attributed to the order of testing. By the time participants completed the sit-ups, they had warmed up, completed five trials of throwing, five trials of kicking, and 2 min of push-ups, all of which likely created fatigue in their abdominal region. Even though testing measures only accumulated about 90 min in total, mental fatigue could have also been a factor. Activities or exercises performed the previous day were not recorded and could have left participants fatigued.

Results indicated that PMC was significantly correlated to both jumping (*r* = 0.350, *p* < 0.01), and push-ups (*r* = 0.410, *p* < 0.01), which indicates individuals who are more skilled have higher perceptions of competence compared to their less-skilled counterparts. Jumping far or having the ability to perform more push-ups can require practice and training over time. If participants know they have not practiced skills like jumping or push-ups (or recently incorporated them into a training regimen), then they may perceive themselves as less likely to be able to do them and vice versa. These findings oppose a previous study by Wang et al. [31] that analyzed the relationship between PMC and the Control Basketball Dribble Test, which found significant correlations between the two variables. A study done by Gu et al. [32] found similar correlations in a younger (e.g., middle childhood) population. The significant correlations between PMC and various MC and HRF measures support the framework created by Stodden and colleagues [9], hypothesizing that the PMC/MC relationships will strengthen over developmental time.

Jump distance was the most correlated variable, with significant correlations to all variables except sit-ups. Similarly, Stodden, Langendorfer, and Roberton [25] found that jumping explained most of the variance (74%) in HRF relative to throwing and kicking. The authors hypothesized that individuals who are skilled in jumping might also partake in other sports/activities that stimulate leg strength and other aspects of physical fitness. The only correlations that existed for PMC were push-ups and jump distance. The push-up test was a measure of upper body strength/endurance. Especially in males, hypertrophy and muscle size that is developed through fitness endeavors is outwardly obvious and potentially important to them regarding physical appearance/attractiveness. It is plausible that this all-male sample could have perceived themselves as stronger, and in turn can do more push-ups, because of their physical features (muscle size) and the exercises they may perform to increase hypertrophy. Jump distance correlates with leg strength; thus, the longer the distance jumped, the greater the leg strength [33]. Males’ leg strength could be due to involvement in other activities/sports/fitness endeavors that inadvertently—or intentionally—increase leg strength. Within the subcomponents that the Physical Self-Perception Profile assessed, physical strength was one. Scoring higher on this specific subcomponent could attribute to participants feeling they are stronger or bigger (in terms of musculature) compared to their peers, so they will perceive themselves as more fit, which helps to explain the correlation that was found between PMC, jump distance, and push-ups.

When MC and HRF were considered as total constructs (Table 3), correlations were non-significant. These findings disagree with other studies that have examined MC and HRF from a “total construct” perspective rather than by individual skills [6,25,26,30,31]. The only significant correlation was between PMC total score and HRF index (*r* = 0.355, *p* < 0.05). It is not surprising that males who have higher physical perceptions of themselves also are highly physically fit. Males tend to value the effects of hypertrophy and muscle strength when exercising, which is evident in their outward appearance [34]. Therefore, for a male with higher perceptions of physical strength and attractiveness, which were constructs within the Physical Self-Perception Profile, it makes sense that the same male would have a physical figure that aligns with his self-perceptions. However, males’ overall PMC was not related to the MC index. Gross MC is not a characteristic that can be observed via physique, unlike physical fitness or strength. It is not presumable that a person has high levels of MC based on their physical appearance compared to the same presumption with physical fitness. The non-association between MC index and PMC could be attributed to the notion that males who are more fit have higher perceptions of themselves compared to males who are highly skilled. Over developmental time, the importance of physical appearance, and inadvertently HRF, may outweigh the importance of MC in males, especially if these motor skills are not utilized or practiced daily. These findings agree with Vedul-Kjelsås et al. [15] who found that PMC was more strongly correlated to HRF than MC in their sample of males.

Table 4 represents the significant predictive effects that MC and PMC had in regard to HRF. Previous studies have demonstrated the association between MC and PMC [3,35,36,37]; however, these findings are limited to child populations and have not been further explored as predictive values of HRF in young adults. The current findings indicate that not only does the performance of motor competency skills influence physical fitness, but also the self-perception of these skills as well. This is important to note, especially in this young adult male population, as these could be key contributors to overall physical health that can be nurtured throughout their development. Within the lens of Stodden et al.’s [9] model, having higher PMC in young adulthood could result in significant physical activity increases later in life. Although physical activity was not assessed in the current study, the significant predictive effects of MC and PMC on HRF give a strong rationale for future research in a similar sample. Similarly, in Table 5, the individual constructs (push-ups, sit-ups, and laps ran) of HRF were significant predictors of males’ PMC. This is a noteworthy finding that gives some insight on what influences a young male adult’s motor competence self-perception. We contend that a more physically fit male may have already mastered some motor skills through sport participation in childhood/adolescence and in turn, developed an increased perception of their motor competence. Those individuals with higher HRF (in regard to push-ups, sit-ups and laps ran) may also believe that they are more motorically competent because of their past physical training or sport participation history. Physical condition, body attractiveness, physical strength, and physical self-worth were all subsections of the Physical Self-Perception Profile, so a more obviously physically fit male will most likely score higher on these sections. Throughout young adulthood and beyond, fostering and improving HRF may positively impact self-perception, creating a stronger relationship over developmental time. Research understanding the dynamic relationship of HRF and PMC in a longitudinal design, between men and women, is warranted.

The study was not without limitations. Females were not assessed in the sample and therefore, findings cannot be generalized to this population. The sample was also comprised exclusively of Physical Education and Coaching majors, so participants’ performance on the variables that were measured may not be representative of the general population. Additionally, the protocol used to collect HRF data relied upon participant counts, and while they were instructed on proper protocol and monitored by the research team, we acknowledge that some error in recorded scores may have occurred. We also could not control for the motivation of the participants. While verbal encouragement was given to all participants during the HRF and MC testing, and participants were instructed to put forth their best effort, it is likely that some participants were inherently more motivated to perform than others. It is plausible that individuals who were less motivated were inherently less physically fit or less inclined to give their best effort. Though our data suggest that the relationship between MC, HRF, and PMC exists in young adulthood, these findings cannot be confirmed without longitudinal data. Furthermore, physical activity was not assessed in the current study, but should be included in future studies to further understand how these health variables influence the trajectory towards overall health. Future research should consider including a young adult sample of both males and females in longitudinal studies of this nature, as the few longitudinal studies that exist only track MC, HRF, and PMC in childhood. It would also be important to examine the MC/HRF/PMC relationship in young adults from fields other than Physical Education and Coaching.

## 5. Conclusions

In summary, our study indicates that there are relationships among MC, HRF, and PMC in a college-aged male sample, but these relationships are expressed differently depending on individual skills and the type of measurement used. As expected, males reported high scores on the Physical Self-Perception Profile questionnaire. Males’ HRF was significantly correlated to their PMC total scores, indicating that men who perceived themselves as being physically competent were actually competent. MC and PMC total scores were predictive indictors of their HRF, and individual measures of HRF were predictive of their PMC total score. Overall, our findings suggest that MC, HRF, and PMC are related in a male college population which can help define the importance of practicing motor competency and maintaining a healthy lifestyle for younger populations.

## Figures and Tables

**Table 1:** Means and standard deviations of physical characteristics and outcome measures.

Variable	M ± SD (*n* = 55)
Age (year)	20.6 ± 1.71
Height (m)	1.78 ± 0.06
Weight (kg)	80.1 ± 12.5
BMI (kg/m^2^)	25.3 ± 3.97
Throw max (kph)	100.7 ± 16.3
Kick max (kph)	74.4 ± 7.93
Jump max (m/ht; %)	1.24% ± 0.17%
MC index	36.6 ± 4.28
Multistage 20-m running test (laps)	47.5 ± 17.7
Push-ups (#)	53.2 ± 18.5
Sit-ups (#)	54.8 ± 17.6
HRF index	51.6 ± 13.2
PMC total	92.2 ± 11.1

Notes: kph = kilometers per hour, BMI = body mass index, MC = motor competence, HRF = health-related fitness, PMC = perceived motor competence score as measured by Physical Self-Perception Profile).

**Table 2:** Pearson’s bivariate correlations between individual variables (*n* = 55).

	1	2	3	4	5	6
1. Throw						
2. Kick	0.352 **					
3. Jump	0.467 **	0.433 *				
4. Laps	0.069	0.070	0.405 **			
5. Sit-ups	0.199	0.144	0.260	0.509 **		
6. Push-ups	0.230	0.345 *	0.601 **	0.201	0.236	
7. PMC Total	0.214	0.242	0.350 *	0.229	0.162	0.410 **

* = statistically significant at *p* < 0.05. ** = statistically significant at *p* < 0.01.

**Table 3:** Pearson’s bivariate correlations between constructs (*n* = 55).

	MC Index	HRF Index
MC Index		
HRF Index	0.276	
PMC Total	0.266	0.355 *

* = statistically significant at *p* < 0.05.

**Table 4:** Predictors of health-related fitness (N = 55).

	*B*	SE *B*	β	R^2^	*F*	*p*
MC Index	0.639	0.427	0.207			
PMC Total	0.384	0.172	0.309			
				0.167	4.60	0.015 *

* = statistically significant at *p* < 0.05.

**Table 5:** Predictors of PMC total score (N = 55).

	*B*	SE *B*	β	R^2^	*F*	*p*
Push-ups	0.213	0.080	0.166			
Sit-ups	−0.014	0.095	−0.023			
Laps	0.100	0.094	0.372			
				0.183	3.37	0.027 *

* = statistically significant at *p* < 0.05.
